# Neonatal diabetes caused by a homozygous *KCNJ11* mutation demonstrates that tiny changes in ATP sensitivity markedly affect diabetes risk

**DOI:** 10.1007/s00125-016-3964-x

**Published:** 2016-04-27

**Authors:** Natascia Vedovato, Edward Cliff, Peter Proks, Varadarajan Poovazhagi, Sarah E. Flanagan, Sian Ellard, Andrew T. Hattersley, Frances M. Ashcroft

**Affiliations:** Department of Physiology, Anatomy and Genetics, University of Oxford, Parks Road, Oxford, OX1 3PT UK; Faculty of Medicine, Nursing and Health Sciences, Monash University, Clayton, VIC Australia; Chengalpattu Medical College, Chengalpattu, Tamil Nadu India; Institute of Biomedical and Clinical Science, University of Exeter Medical School, Exeter, UK

**Keywords:** ATP-sensitive potassium channel, ATP sensitivity, *KCNJ11*, Neonatal diabetes, Type 2 diabetes

## Abstract

**Aims/hypothesis:**

The pancreatic ATP-sensitive potassium (K_ATP_) channel plays a pivotal role in linking beta cell metabolism to insulin secretion. Mutations in K_ATP_ channel genes can result in hypo- or hypersecretion of insulin, as in neonatal diabetes mellitus and congenital hyperinsulinism, respectively. To date, all patients affected by neonatal diabetes due to a mutation in the pore-forming subunit of the channel (Kir6.2, *KCNJ11*) are heterozygous for the mutation. Here, we report the first clinical case of neonatal diabetes caused by a homozygous *KCNJ11* mutation.

**Methods:**

A male patient was diagnosed with diabetes shortly after birth. At 5 months of age, genetic testing revealed he carried a homozygous *KCNJ11* mutation, G324R, (Kir6.2-G324R) and he was successfully transferred to sulfonylurea therapy (0.2 mg kg^−1^ day^−1^). Neither heterozygous parent was affected. Functional properties of wild-type, heterozygous and homozygous mutant K_ATP_ channels were examined after heterologous expression in *Xenopus* oocytes.

**Results:**

Functional studies indicated that the Kir6.2-G324R mutation reduces the channel ATP sensitivity but that the difference in ATP inhibition between homozygous and heterozygous channels is remarkably small. Nevertheless, the homozygous patient developed neonatal diabetes, whereas the heterozygous parents were, and remain, unaffected. Kir6.2-G324R channels were fully shut by the sulfonylurea tolbutamide, which explains why the patient’s diabetes was well controlled by sulfonylurea therapy.

**Conclusions/interpretation:**

The data demonstrate that tiny changes in K_ATP_ channel activity can alter beta cell electrical activity and insulin secretion sufficiently to cause diabetes. They also aid our understanding of how the Kir6.2-E23K variant predisposes to type 2 diabetes.

**Electronic supplementary material:**

The online version of this article (doi:10.1007/s00125-016-3964-x) contains peer-reviewed but unedited supplementary material, which is available to authorised users.

## Introduction

Neonatal diabetes is a rare genetic disorder characterised by diabetes that presents within the first 6 months of life and which may be either permanent (PNDM) or transient (TNDM). Approximately 50% of cases of neonatal diabetes are due to gain-of-function mutations in the genes that encode the pore-forming (*KCNJ11*, encoding the inwardly rectifying potassium channel Kir6.2) or regulatory (*ABCC8*, encoding the sulfonylurea receptor [SUR] 1) subunits of the ATP-sensitive potassium (K_ATP_) channel [1–3]. In ∼30% of these patients, neurological symptoms such as developmental delay and muscle hypotonia are also found, a condition termed intermediate DEND (iDEND) syndrome [2]. About 3% of patients also experience epilepsy (DEND syndrome, defined as developmental delay, epilepsy and neonatal diabetes). K_ATP_ channel mutations are also associated with diabetes that presents in later life [4–6], and a common polymorphism in *KCNJ11* (E23K) confers an enhanced risk of type 2 diabetes [7–9].

The K_ATP_ channel plays a fundamental role in multiple tissues by coupling cell metabolism to electrical activity and thereby cell function. In pancreatic beta cells, it links plasma glucose levels to insulin secretion, and in neurons it modulates neuronal activity and neurotransmitter release [10]. Metabolic regulation is mediated via changes in intracellular adenine nucleotide levels, with ATP closing the channel by binding to Kir6.2 and Mg nucleotides, stimulating channel opening by interaction with SUR1 [11–13]. In the beta cell, K_ATP_ channel closure leads to electrical activity, calcium influx and thereby insulin granule exocytosis. Activating K_ATP_ channel mutations cause neonatal diabetes by impairing channel inhibition by MgATP and thereby preventing glucose-induced insulin secretion [1–3, 10]. Compared with PNDM (or TNDM), DEND and iDEND syndromes are associated with functionally more severe mutations that cause a greater reduction in ATP sensitivity and thus affect neurons as well as beta cells [10]. It is believed that a very small reduction in ATP inhibition underlies the ability of the Kir6.2-E23K variant to enhance type 2 diabetes risk [14–16]. However, this has been difficult to prove conclusively.

To date, all patients with neonatal diabetes due to mutations in the gene encoding Kir6.2 have been heterozygous for the mutation. Identification of a homozygous mutation causing recessive neonatal diabetes, however, would clearly define the extent of the reduction in channel ATP sensitivity that is sufficient to cause neonatal diabetes and also help determine whether the small decrease in ATP sensitivity caused by the Kir6.2-E23K variant is enough to account for the increased type 2 diabetes risk.

## Methods

### Molecular genetics

Genomic DNA was extracted from peripheral leucocytes using standard procedures. The coding regions and conserved splice sites of the *ABCC8* and *KCNJ11* genes were amplified by PCR and the resulting amplicons sequenced using the BigDye Terminator Cycle v3.1 Sequencing Kit (Applied Biosystems, Warrington, UK). The products were analysed on an ABI 3730 capillary sequencer (Applied Biosystems) and compared with the reference sequences (NM_000525.3 and NM_000352.3) using Mutation Surveyor version 3.24 software (SoftGenetics, State College, PA, USA).

### Molecular biology and oocyte preparation

We used human *KCNJ11* (GenBank NM000525, with E23 and I337 [except where stated]) and rat *Abcc8* (GenBank L40624). Site-directed mutagenesis of *KCNJ11* was performed using the QuikChange XL system (Stratagene, La Jolla, CA, USA). *KCNJ11* and *Abcc8* mRNAs were prepared using the mMESSAGE mMACHINE large-scale in vitro transcription kit (Ambion, Austin, TX, USA), as described previously [17]. Defolliculated *Xenopus laevis* oocytes were injected with 0.8 ng wild-type (WT) (or mutant) *KCNJ11* mRNA and 4 ng *Abcc8* mRNA, and incubated in Barth’s solution at 18°C for 1–4 days. To simulate the heterozygous state of unaffected heterozygous carriers, we coinjected a 1:1 mixture of mutant and WT *KCNJ11*, together with *Abcc8*. The resulting channel population (referred to here as heterozygous [het]G324R) will contain a variable number of mutant subunits (between zero and four) in the Kir6.2 tetramer.

### Electrophysiology

Whole oocyte currents were recorded using a two-electrode voltage clamp (GeneClamp 500B; Molecular Devices, Sunnyvale, CA, USA), in response to 500 ms voltage steps of ±20 mV from a holding potential of -10 mV. Data were acquired at 4 kHz, after online filtering at 0.5 kHz, using a 1440A Digidata interface (Molecular Devices), computer controlled by pCLAMP 10 software (Molecular Devices). Oocytes were continuously perfused with (in mmol/l) 90 KCl, 1 MgCl_2_, 1.8 CaCl_2_ and 5 HEPES (adjusted to pH 7.4 with KOH), supplemented with sodium azide (3 mmol/l) or tolbutamide (0.5 mmol/l, diluted from a 50 mmol/l stock solution in DMSO), as indicated.

Macroscopic currents were recorded at -60 mV from giant excised inside-out patches, using an Axopatch 200B amplifier (Molecular Devices), filtered at 1 kHz and sampled at 10 kHz with a Digidata 1322A A/D-D/A driven by pCLAMP 9 software (Molecular Devices). The pipette (extracellular) solution contained (in mmol/l) 140 KCl, 1.2 MgCl_2_, 2.6 CaCl_2_ and 10 HEPES (adjusted to pH 7.4 with KOH). The bath (cytoplasmic) solution contained (in mmol/l) 107 KCl, 2 MgCl_2_, 1 CaCl_2_, 10 EGTA, 10 HEPES (adjusted to pH 7.2 with KOH) and MgATP, as indicated.

MgATP concentration–response curves were individually fitted with the Hill equation: I/I_C_ = 1/(1 + ([MgATP]/IC_50_)^h^, where [MgATP] indicates the MgATP concentration, IC_50_ is the MgATP concentration that causes half-maximal block, I_C_ and I are the currents in the absence and presence of nucleotide, respectively, and h is the Hill coefficient. To correct for K_ATP_ current rundown, I_C_ was taken as the mean of the current in nucleotide-free solution before and after each ATP application. In addition, only patches in which the response to 100 μmol/l MgATP did not change during the course of the experiment were included in the analysis. Data were analysed with Clampfit (pCLAMP 9 or 10; Molecular Devices) and Origin 7.0 (OriginLab, Northampton, MA, USA). Results are given as mean ± SEM of *n* measurements from at least two independent batches of oocytes.

## Results

### Clinical and genetic data

A male patient of Indian ethnicity was diagnosed with diabetes at 11 weeks of age (birthweight 2,700 g at term). At the age of 5 months, following a referral for genetic testing, sequence analysis identified a novel homozygous missense mutation, p.G324R (c.970G>A), in the *KCNJ11* gene (referred to here as Kir6.2-G324R). Sequence analysis of the *ABCC8* gene or insulin gene did not identify a mutation.

Following genetic diagnosis, the patient transferred from insulin (2.1 U kg^−1^ day^−1^) to glibenclamide (0.2 mg kg^−1^ day^−1^), with an improvement in HbA_1c_ (8.4% pre-transfer to 5.6% post-transfer [68 mmol/mol to 38 mmol/mol]). The patient is currently 4.5 years of age and is not requiring any treatment. The age at remission of diabetes is unknown.

Both parents, who are not diabetic at the age of 29 years and 34 years, respectively, were heterozygous for the Kir6.2-G324R mutation. Samples from the unaffected grandparents were not available for testing.

### Metabolic regulation of the K_ATP_ channel

**Fig. 1:**
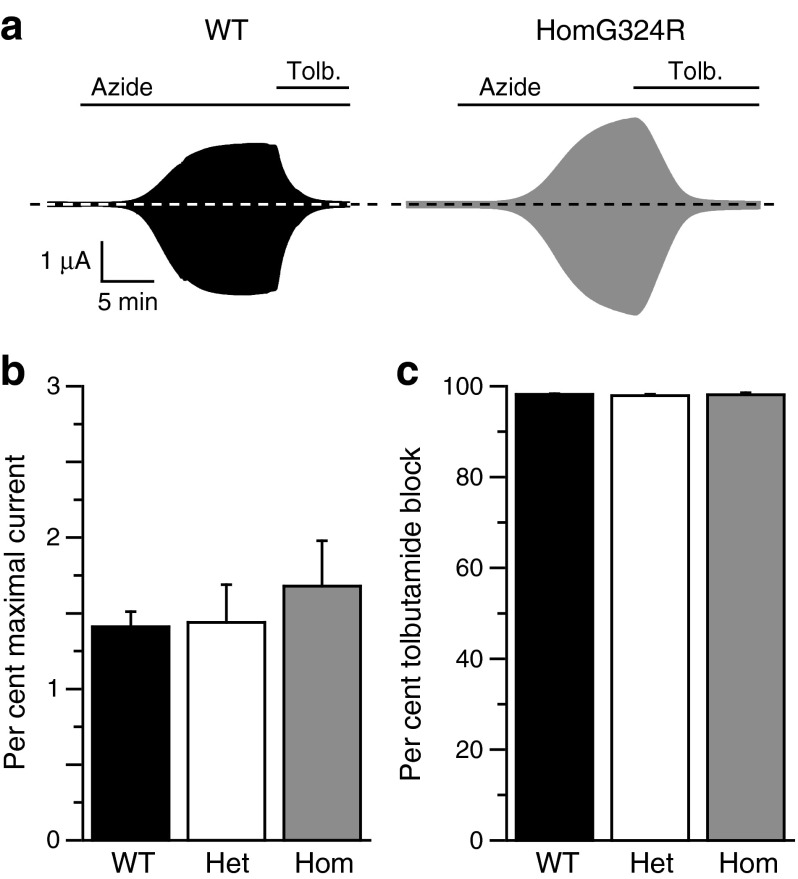
Metabolic activation of WT and mutant K_ATP_ channels. (**a**) Representative whole-cell currents recorded from oocytes expressing WT or homG324R channels in response to 500 ms voltage steps of ±20 mV from a holding potential of −10 mV. Sodium azide (azide, 3 mmol/l) and tolbutamide (tolb., 0.5 mmol/l) were added as indicated. (**b**) Current in control solution for WT (*n* = 13), hetG324R (het; *n* = 9) and homG324R (hom; *n* = 10) expressed as a percentage of that in the presence of 3 mmol/l sodium azide (per cent maximal current). (**c**) Mean tolbutamide block for WT (98.2 ± 0.2%, *n* = 7), hetG324R (het; 97.9 ± 0.3%, *n* = 6) and homG324R (hom; 98.1 ± 0.4%, *n* = 7) channels (measured in the presence of sodium azide)

We analysed the effects of the Kir6.2-G324R mutation on metabolic regulation of the K_ATP_ channel by measuring whole-cell currents. When WT K_ATP_ channels are expressed in *Xenopus* oocytes they are normally closed, due to the high intracellular ATP concentration. However, they can be opened by lowering the intracellular ATP concentration using a metabolic inhibitor such as sodium azide (Fig. 1a). Mutations that reduce the channel ATP sensitivity normally increase the whole-cell current in control solution, reflecting the fact that they are less blocked by the resting intracellular ATP concentration [10, 18]. Homozygous Kir6.2-G324R/SUR1 currents (homG324R) were not appreciably different from WT either in control solution or in the presence of azide (Fig. 1a).

To control for variability between different oocytes (and different batches of oocytes), we expressed the resting current in control solution as a percentage of the maximal current recorded in the presence of 3 mmol/l sodium azide. Mean data are given in Fig. 1b. There was no difference between WT and heterozygous Kir6.2-G324R/SUR1 currents (hetG324R), which may explain why heterozygous carriers did not develop neonatal diabetes. HomG324R currents were slightly greater than either WT or hetG324R currents, although this difference did not reach significance.

The K_ATP_ channel inhibitor tolbutamide (0.5 mmol/l) blocked all three types of channel by ∼98% (Fig. 1c). This explains why the neonatal diabetes of homG324R patients can be controlled by sulfonylurea therapy. Indeed, homG324R channels were blocked by tolbutamide as efficiently as WT channels, which suggests the patient may be at somewhat higher risk of hypoglycaemia than patients with more strongly activating K_ATP_ channel mutations that are less sensitive to sulfonylureas.

### ATP sensitivity

**Fig. 2:**
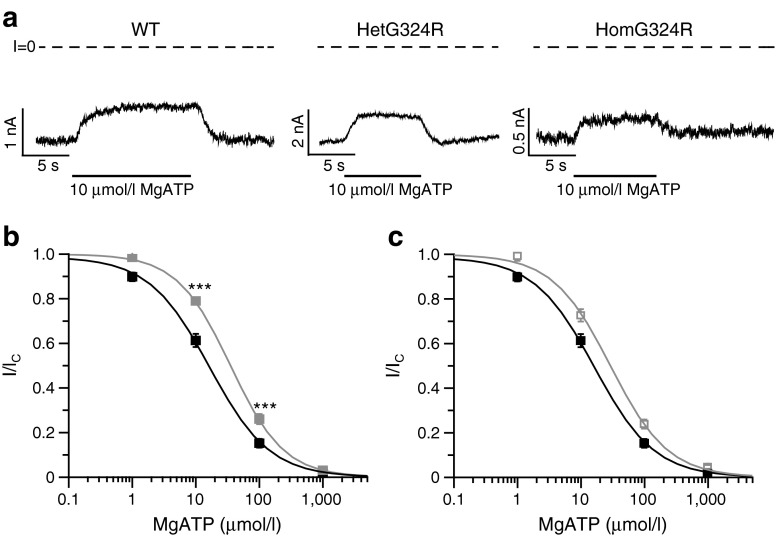
MgATP inhibition of K_ATP_ channels is reduced by the Kir6.2-G324R mutation. (**a**) Representative currents recorded at −60 mV from inside-out patches excised from oocytes expressing WT or mutant K_ATP_ channels. The dashed line indicates the zero current level. MgATP (10 μmol/l) was applied as indicated. (**b**, **c**) Relationship between K_ATP_ current and MgATP concentration for WT (black squares), homG324R (grey filled squares; **b**) and hetG324R (grey empty squares; **c**) channels. Current in the presence of nucleotide (I) is expressed as a fraction of that in its absence (I_C_). The curves are the best fit to the Hill equation. ***p < 0.001 compared with WT (*t* test)

**Fig. 3:**
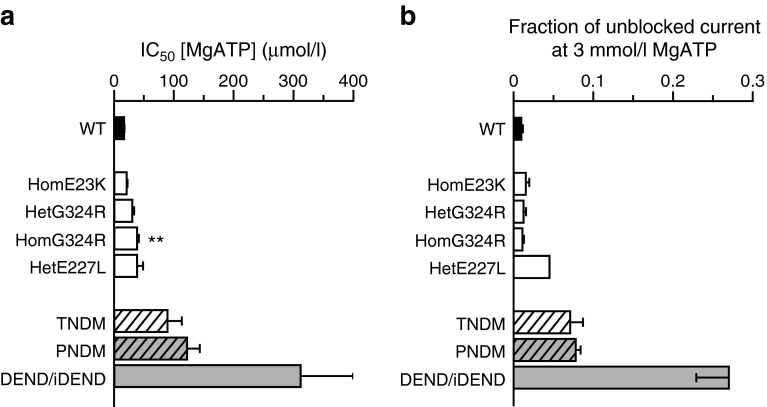
Comparison of ATP sensitivities. (**a**) IC_50_ for MgATP inhibition and (**b**) fraction of unblocked current at 3 mmol/l MgATP for the indicated channels: WT (*n* = 13), homE23K (*n* = 5), hetG324R (*n* = 6), homG324R (*n* = 8), hetE227L (*n* = 7). Mean values for TNDM (white hatched bars), PNDM (grey hatched bars) and DEND/iDEND (grey bars) channels are given for comparison (for references, see ESM Fig. 1). Data are mean ± SEM. **p < 0.01 compared with WT (*t* test)

We examined the ATP sensitivity of WT and mutant channels in the presence of 2 mmol/l Mg^2+^, to approximate the physiological condition (Figs 2, 3). WT channels were half maximally blocked (IC_50_) by 18 ± 2 μmol/l MgATP (*n* = 13). The ATP sensitivity of homG324R channels was slightly but significantly smaller than WT (IC_50_ = 38 ± 3 μmol/l, *n* = 8; *p* < 0.01 vs WT) (Figs 2b, 3a). The ATP sensitivity of hetG324R channels was also reduced but the difference was not significant (IC_50_ = 30 ± 3 μmol/l, *n* = 6 (Figs 2c, 3a). For comparison, the IC_50_ was 21 ± 2 μmol/l (*n* = 5) for K_ATP_ channels containing the Kir6.2-K23 variant.

The K_ATP_ current amplitude at physiological ATP concentrations is critical, as this will determine the beta cell resting potential and thus the clinical phenotype. At 3 mmol/l MgATP, a concentration within the physiological range, the percentage of unblocked current was 1.1 ± 0.2% (*n* = 8) for homG324R channels and 1.2 ± 0.3% (*n* = 6) for hetG324R channels, compared with 0.8 ± 0.2% (*n* = 13) for WT channels. These differences were not significant (Fig. 3b).

We observed no significant difference in homG324R and WT current magnitudes in control solution in excised patches, suggesting the Kir6.2-G324R mutation does not alter membrane trafficking.

## Discussion

The difference in ATP sensitivity between recombinant K_ATP_ channels homomeric for the Kir6.2-G324R mutation and pseudo-heterozygous hetG324R channels is strikingly small: the IC_50_ differs by only 8 μmol/l; yet, the proband homozygous for the G324R mutation developed neonatal diabetes, whereas his heterozygous parents were unaffected. Thus, tiny changes in ATP sensitivity can cause disease. This is not surprising, however, given that very small differences in K_ATP_ current magnitude can lead to marked differences in beta cell membrane potential and electrical activity (and thus insulin secretion), if they occur close to the action potential threshold, where the electrical resistance of the beta cell membrane is very high [19]. This is because the K_ATP_ current dominates the beta cell resting membrane potential. For a detailed explanation of how the beta cell membrane potential (and electrical activity) can be exquisitely sensitive to tiny changes in K_ATP_ channel activity, see the review by Ashcroft and Rorsman [20].

**Fig. 4:**
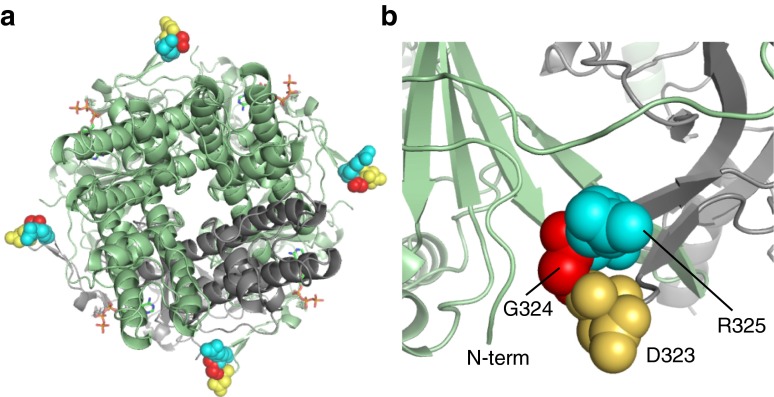
Molecular model of the Kir6.2 tetramer [19]. (**a**) Top view from the extracellular side, showing the position of G324 (red), D323 (yellow) and R325 (cyan). ATP (orange sticks) is shown docked into its putative binding site. (**b**) Detail of two adjacent Kir6.2 subunits (one green, one grey). The first 32 amino acids are not included in the model so N-term denotes residue 32

The ATP sensitivity measured for homG324R channels (38 μmol/l) lies within the lower range of that reported for heterozygous *KCNJ11* mutations associated with PNDM (range 34–273 μmol/l) or TNDM (range 27–213 μmol/l) (electronic supplementary material [ESM] Fig. 1). For comparison, the mean IC_50_ value obtained for DEND/iDEND mutations is 312 ± 89 μmol/l (*n* = 11), for PNDM it is 122 ± 21 μmol/l (*n* = 10), for TNDM it is 89 ± 22 μmol/l (*n* = 8) and for WT channels it is ∼15 μmol/l (Fig. 3a). Thus, the more severe clinical phenotype (DEND/iDEND) is associated with a greater reduction in ATP sensitivity. Interestingly, the Kir6.2-E227L mutation that causes a variable disease phenotype, where some patients do not develop diabetes until early adult life or only during pregnancy, causes a much smaller reduction in ATP sensitivity [5], comparable with that observed for the homG324R mutation (Fig. 3a). Similarly, the percentage of unblocked current at 3 mmol/l MgATP was greater for mutations associated with a more severe clinical phenotype (Fig. 4b, ESM Fig. 1b).

### Mechanism of action

In a molecular model of Kir6.2 (Fig. 4), G324 lies far from the putative ATP-binding site [21] and thus its mutation is unlikely to impair ATP binding directly. Rather, it is expected to reduce ATP inhibition indirectly, either by allosterically impairing ATP binding or by enhancing the intrinsic open probability of the channel [18]. Importantly, the adjacent residue, D323, forms an ion pair with K1322 in SUR2A [22], and may be predicted to form a similar interaction with the equivalent residue in SUR1 (K1355). Furthermore, mutation of D323 to the positively charged amino acid lysine, which would disrupt the Kir6.2–SUR1 pair, causes PNDM, reducing the channel ATP sensitivity to 129 μmol/l in the heterozygous state and to 408 μmol/l in the homozygous state [23]. Potentially, therefore, the G324R mutation, which also introduces a positive charge, might disrupt the adjacent Kir6.2–SUR1 ion pair.

### Relevance to type 2 diabetes

A common polymorphism (E23K) in *KCNJ11* is associated with increased susceptibility to type 2 diabetes [8]. Although the increase in risk is relatively small (OR 1.18), it is highly significant. Functional studies have shown that in people with normal glucose tolerance, the K variant is associated with a marked (40%) reduction in insulin secretion in response to an oral or intravenous glucose challenge [9].

The molecular basis of the increased predisposition to type 2 diabetes remains unclear, as the reported effect of the mutation on K_ATP_ channel activity differs between different laboratories. Most studies have found the K variant is associated with a very small reduction in sensitivity to ATP inhibition [9, 14]. However, others have reported that it enhances ATP sensitivity [24] or is without effect [16]. Similarly, increased activation by MgADP of Kir6.2-K23 channels was observed in one study [25], but not found in another [16]. Enhanced activation of Kir6.2-K23 channels by long-chain acyl-CoAs has also been observed [24]. A further complication is that the E23K polymorphism is linked both to a second polymorphism in *KCNJ11* (I337V) and to a polymorphism in *ABCC8* (A1369S) [26], and a recent study suggested that it was the SUR1-A1369S variant (not the Kir6.2-E23K polymorphism) that was the critical determinant of the difference in K_ATP_ channel ATP sensitivity [16]. This may be attributable to the increased ability of MgATP (via its hydrolysis) to activate the SUR1-A1369 variant [27].

Our finding that a tiny difference in ATP sensitivity—from 30 μmol/l (hetG324R) to 38 μmol/l (homG324R)—is sufficient to cause neonatal diabetes may help to resolve this conundrum, as it predicts an even smaller reduction in ATP sensitivity might be sufficient to predispose to type 2 diabetes. Given the usual biological variability, such a small difference may be hard to detect above the background noise. It is worth noting that genetic studies required >1,000 patients to detect a significant increase in risk [8]—a sample size not possible in electrophysiological experiments. In addition, differences in cell type, the amount of phosphatidylinositol 4,5-bisphosphate (PIP_2_) in the membrane, and methodology (such as the time after patch excision that ATP sensitivity is measured) may contribute to the small differences seen between laboratories.

Our data help explain why patients with the Kir6.2-E23K polymorphism do not develop diabetes at birth: the reduction in ATP sensitivity is simply too small. They also clarify why it has been so difficult to detect differences in K_ATP_ channel function due to the Kir6.2-E23K/SUR1-A1369S mutations, as they reveal that the difference in ATP sensitivity between mutations causing neonatal diabetes (38 μmol/l) or not (30 μmol/l) is extremely small. Finally, they suggest the unaffected heterozygous Kir6.2-G324R parents may be at increased risk of developing type 2 diabetes in later life. Whether they do so may depend on their genetic background and environmental factors such as an obesogenic lifestyle.

## Electronic supplementary material

Below is the link to the electronic supplementary material.

ESM Fig. 1 (PDF 131 kb)

## Abbreviations

DEND: Developmental delay, epilepsy and neonatal diabetes
Het: Heterozygous mutation, e.g. hetG324R
Hom: Homozygous mutation, e.g. homG324R
iDEND: Intermediate DEND
K_ATP_ channel: ATP-sensitive potassium channel
Kir: Potassium inward rectifier
PNDM: Permanent neonatal diabetes mellitus
SUR: Sulfonylurea receptor
TNDM: Transient neonatal diabetes mellitus
WT: Wild-type

## References

[1] Gloyn AL, Pearson ER, Antcliff JF (2004). Activating mutations in the gene encoding the ATP-sensitive potassium-channel subunit Kir6.2 and permanent neonatal diabetes. N Engl J Med.

[2] Hattersley AT, Ashcroft FM (2005). Activating mutations in Kir6.2 and neonatal diabetes: new clinical syndromes, new scientific insights, and new therapy. Diabetes.

[3] Flanagan SE, Clauin S, Bellanné-Chantelot C (2009). Update of mutations in the genes encoding the pancreatic beta-cell K_ATP_ channel subunits Kir6.2 (*KCNJ11*) and sulfonylurea receptor 1 (*ABCC8*) in diabetes mellitus and hyperinsulinism. Hum Mutat.

[4] Yorifuji T, Nagashima K, Kurokawa K (2005). The C42R mutation in the Kir6.2 (*KCNJ11*) gene as a cause of transient neonatal diabetes, childhood diabetes, or later-onset, apparently type 2 diabetes mellitus. J Clin Endocrinol Metab.

[5] D'Amato E, Tammaro P, Craig TJ (2008). Variable phenotypic spectrum of diabetes mellitus in a family carrying a novel *KCNJ11* gene mutation. Diabet Med.

[6] Liu L, Nagashima K, Yasuda T (2013). Mutations in *KCNJ11* are associated with the development of autosomal dominant, early-onset type 2 diabetes. Diabetologia.

[7] Sakura H, Wat N, Horton V, Millns H, Turner RC, Ashcroft FM (1996). Sequence variations in the human Kir6.2 gene, a subunit of the ß-cell ATP-sensitive K-channel: no association with NIDDM in white Caucasian subjects or evidence of abnormal function when expressed *in vitro*. Diabetologia.

[8] Gloyn AL, Weedon MN, Owen KR (2003). Large-scale association studies of variants in genes encoding the pancreatic beta-cell K_ATP_ channel subunits Kir6.2 (*KCNJ11*) and SUR1 (*ABCC8*) confirm that the *KCNJ11* E23K variant is associated with type 2 diabetes. Diabetes.

[9] Villareal DT, Koster JC, Robertson H (2009). Kir6.2 variant E23K increases ATP-sensitive K^+^ channel activity and is associated with impaired insulin release and enhanced insulin sensitivity in adults with normal glucose tolerance. Diabetes.

[10] Ashcroft FM (2007). The Walter B. Cannon physiology in perspective lecture, 2007. ATP-sensitive K^+^ channels and disease: from molecule to malady. Am J Physiol Endocrinol Metab.

[11] Cook DL, Hales CN (1984). Intracellular ATP directly blocks K^+^ channels in pancreatic B cells. Nature.

[12] Nichols CG, Shyng SL, Nestorowicz A (1996). Adenosine diphosphate as an intracellular regulator of insulin secretion. Science.

[13] Tucker SJ, Gribble FM, Zhao C, Trapp S, Ashcroft FM (1997). Truncation of Kir6.2 produces ATP-sensitive K^+^ channels in the absence of the sulphonylurea receptor. Nature.

[14] Schwanstecher C, Meyer U, Schwanstecher M (2002). K_IR_6.2 polymorphism predisposes to type 2 diabetes by inducing overactivity of pancreatic beta-cell ATP-sensitive K^+^ channels. Diabetes.

[15] Riedel MJ, Steckley DC, Light PE (2005). Current status of the E23K Kir6.2 polymorphism: implications for type-2 diabetes. Hum Genet.

[16] Hamming KSC, Soliman D, Matemisz LC (2009). Coexpression of the type 2 diabetes susceptibility gene variants *KCNJ11* E23K and *ABCC8* S1369A alter the ATP and sulfonylurea sensitivities of the ATP-sensitive K^+^ channel. Diabetes.

[17] Gribble FM, Ashfield R, Ammälä C, Ashcroft FM (1997). Properties of cloned ATP-sensitive K^+^ currents expressed in *Xenopus* oocytes. J Physiol Lond.

[18] Proks P, Antcliff JF, Lippiat J, Gloyn AL, Hattersley AT, Ashcroft FM (2004). Molecular basis of Kir6.2 mutations associated with neonatal diabetes or neonatal diabetes plus neurological features. Proc Natl Acad Sci U S A.

[19] Tarasov AI, Welters HJ, Senkel S (2006). A Kir6.2 mutation causing neonatal diabetes impairs electrical activity and insulin secretion from INS-1 beta-cells. Diabetes.

[20] Ashcroft FM, Rorsman P (2004). Type 2 diabetes mellitus: not quite exciting enough?. Hum Mol Genet.

[21] Antcliff JF, Haider S, Proks P, Sansom MSP, Ashcroft FM (2005). Functional analysis of a structural model of the ATP-binding site of the K_ATP_ channel Kir6.2 subunit. EMBO J.

[22] Lodwick D, Rainbow RD, Rubaiy HN, Al Johi M, Vuister GW, Norman RI (2014). Sulfonylurea receptors regulate the channel pore in ATP-sensitive potassium channels via an intersubunit salt bridge. Biochem J.

[23] Tarasov AI, Girard CA, Larkin B (2007). Functional analysis of two Kir6.2 (*KCNJ11*) mutations, K170T and E322K, causing neonatal diabetes. Diabetes Obes Metab.

[24] Riedel MJ, Boora P, Steckley D, de Vries G, Light PE (2003). Kir6.2 polymorphisms sensitize beta-cell ATP-sensitive potassium channels to activation by acyl CoAs: a possible cellular mechanism for increased susceptibility to type 2 diabetes?. Diabetes.

[25] Schwanstecher C, Neugebauer B, Schulz M, Schwanstecher M (2002). The common single nucleotide polymorphism E23K in K_IR_6.2 sensitizes pancreatic beta-cell ATP-sensitive potassium channels toward activation through nucleoside diphosphates. Diabetes.

[26] Florez JC, Burtt N, de Bakker PIW (2004). Haplotype structure and genotype-phenotype correlations of the sulfonylurea receptor and the islet ATP-sensitive potassium channel gene region. Diabetes.

[27] Fatehi M, Raja M, Carter C, Soliman D, Holt A, Light PE (2012). The ATP-sensitive K^+^ channel *ABCC8* S1369A type 2 diabetes risk variant increases MgATPase activity. Diabetes.

