# Supplementary material for: Neonatal diabetes caused by a homozygous KCNJ11 mutation demonstrates that tiny changes in ATP sensitivity markedly affect diabetes risk
Source: Diabetologia. 2016 Apr 27;59:1430–6. doi: 10.1007/s00125-016-3964-x (PMC4901145; doi:10.1007/s00125-016-3964-x)
Supplement: Supplementary file 1 — (PDF 131 kb) [file 125_2016_3964_MOESM1_ESM.pdf]

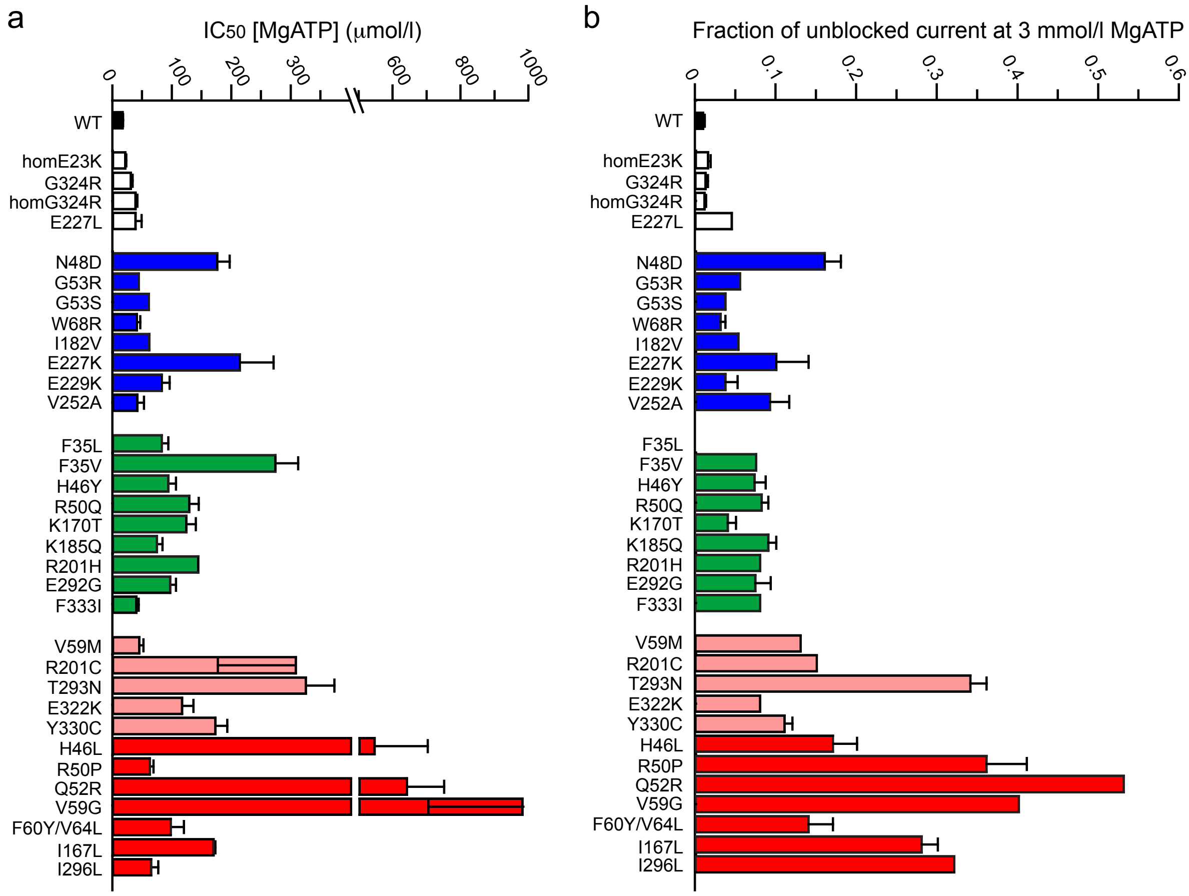

ESM Fig. 1 Comparison of ATP sensitivities. (**a**) IC_{50} for MgATP inhibition, and (**b**) the fraction of unblocked current at 3 mmol/l MgATP for WT and the indicated Kir6.2 mutant channels. Homomeric channels (E23K, G324R) are indicated by 'hom'; all other data are from pseudo-heterozygous K_{ATP} channels. White bars, mutations causing/predisposing to diabetes in later life (see text); blue bars, TNDM; green bars, PNDM; pink bars, iDEND syndrome; red bars, DEND syndrome. Data for mutations other than G324R and E23K are taken from previous studies (see below [1-16]). Note the IC_{50} values for some DEND mutations (a) do not adequately reflect the mutation/disease severity, as these channels exhibit a large pedestal of unblocked current at physiological ATP levels (as seen in b). Data are mean±SEM.

References

1. D'Amato E, Tammaro P, Craig TJ, et al (2008) Variable phenotypic spectrum of diabetes

mellitus in a family carrying a novel *KCNJ11* gene mutation. Diabet Med 25:651-656

2. Gloyn AL, Reimann F, Girard C, et al (2005) Relapsing diabetes can result from moderately activating mutations in *KCNJ11*. Hum Mol Genet 14:925-934
3. Girard CA, Shimomura K, Proks P, et al (2006) Functional analysis of six Kir6.2 (*KCNJ11*) mutations causing neonatal diabetes. Pflugers Arch 453:323-332
4. Mlynarski W, Tarasov AI, Gach A, et al (2007) Sulfonylurea improves CNS function in a case of intermediate DEND syndrome caused by a mutation in *KCNJ11*. Nat Clin Pract Neurol 3:640-645
5. Männikkö R, Jefferies C, Flanagan SE, et al (2010) Interaction between mutations in the slide helix of Kir6.2 associated with neonatal diabetes and neurological symptoms. Hum Mol Genet 19:963-972
6. Männikkö R, Stansfeld PJ, Ashcroft AS, et al (2011) A conserved tryptophan at the membrane-water interface acts as a gatekeeper for Kir6.2/SUR1 channels and causes neonatal diabetes when mutated. J Physiol 589:3071-3083
7. Proks P, Girard C, Ashcroft FM (2005) Functional effects of *KCNJ11* mutations causing neonatal diabetes: enhanced activation by MgATP. Hum Mol Genet 14:2717-2726
8. Proks P, Girard C, Haider S, et al (2005) A gating mutation at the internal mouth of the Kir6.2 pore is associated with DEND syndrome. EMBO Rep 6:470-475
9. Proks P, Girard C, Baevre H, et al (2006) Functional effects of mutations at F35 in the NH_2-terminus of Kir6.2 (*KCNJ11*), causing neonatal diabetes, and response to sulfonylurea therapy. Diabetes 55:1731-1737
10. Shimomura K, Girard CA, Proks P, et al (2006) Mutations at the same residue (R50) of Kir6.2 (*KCNJ11*) that cause neonatal diabetes produce different functional effects. Diabetes 55:1705-1712
11. Shimomura K, Hörster F, de Wet H, et al (2007) A novel mutation causing DEND syndrome: a treatable channelopathy of pancreas and brain. Neurology 69:1342-1349
12. Shimomura K, Flanagan SE, Zadek B, et al (2009) Adjacent mutations in the gating loop of Kir6.2 produce neonatal diabetes and hyperinsulinism. EMBO Mol Med 1:166-177
13. Shimomura K, de Nanclares GP, Foutinou C, et al (2010) The first clinical case of a mutation at residue K185 of Kir6.2 (*KCNJ11*): a major ATP-binding residue. Diabet Med 27:225-229
14. Tammaro P, Girard C, Molnes J, et al (2005) Kir6.2 mutations causing neonatal diabetes

provide new insights into Kir6.2-SUR1 interactions. EMBO J 24:2318-2330

15. Tammaro P, Proks P, Ashcroft FM (2006) Functional effects of naturally occurring *KCNJ11* mutations causing neonatal diabetes on cloned cardiac K_{ATP} channels. J Physiol 571:3-14
16. Tarasov AI, Girard CA, Larkin B, et al (2007) Functional analysis of two Kir6.2 (*KCNJ11*) mutations, K170T and E322K, causing neonatal diabetes. Diabetes Obes Metab 9:46-55
